# The CellFX Percutaneous Electrode System for Nanosecond Pulsed Field Ablation

**DOI:** 10.1089/bioe.2024.0015

**Published:** 2024-09-16

**Authors:** Richard Nuccitelli, Jeffrey Litt, Kevin Moss, Dale Hinman, Ilya Getsin, Darrin Uecker, Bill Stoffregen, William A. Knape

**Affiliations:** ^1^ Pulse Biosciences, Inc., Hayward, California, USA.; ^2^ Northstar Preclinical and Pathology Services, Lake Elmo, Minnesota, USA.

**Keywords:** percutaneous electrode, CellFX, nanosecond electric pulses, ablation, regulated cell death, apoptosis, nsPFA, Nanosecond pulsed field ablation

## Abstract

The purpose of this study was to demonstrate the safety and performance of the CellFX Percutaneous Electrode for delivering nanosecond pulsed field ablation (nsPFA) energy to soft tissues. Three different porcine tissue types were treated, namely, liver, kidney, and skeletal muscle, at treatment levels of three times greater than clinical treatment levels. The histological characteristics of the ablation zone for each of these tissues compared with that of radiofrequency (RF) ablation on day 0 and 2 days post-treatment. Ablation zone dimensions were measured during gross necropsy after tetrazolium chloride staining and compared between the nsPFA and RF groups at 2 days post-ablation. The CellFX system successfully achieved ablation and necrosis of all treatment sites in all target tissues. No evidence of thermal effects or collagen degeneration was found at any of the nsPFA treatment sites. Overall systemic tolerability was evidenced by the absence of clinically significant changes in urinalysis and serum chemistry before and after treatments.

## Introduction

The CellFX^®^ Percutaneous Electrode System is a proprietary platform for delivering nanosecond pulsed field ablation (nsPFA) technology. This system delivers ultrafast, nonthermal, nanosecond (billionth of a second) energy pulses to targeted tissue. When energy is applied to targeted tissue, nsPFA pulses enter cells and disrupt the function of internal organelles, including mitochondria and endoplasmic reticulum.^1^ The cascade of intracellular disruption leads to regulated or programmed cell death (RCD). RCD is a process exhibited by all the cells in the body when they reach the end of their useful life or are unable to restore cellular homeostasis.^2^,^3^ This pathway is well documented^3^ and includes an increase in intracellular Ca^2+^,^4^ generation of reactive oxygen species,^5^ DNA fragmentation,^6^ caspase 3 activation^7^ to hydrolyze proteins, release of cytochrome C from mitochondria,^8^ and the translocation of calreticulin to the plasma membrane.^3^

The main goal of this study was to demonstrate the safety and performance of the CellFX Percutaneous Electrode System in porcine tissue at day 0 and day 2 and compare the tissue histology with treatments created with a radiofrequency (RF) ablation system using bipolar RF energy.

## Materials and Methods

### Animals

Six female Yorkshire swine were included in this study weighing between 59 and 80 kg. All treatments were conducted on animals under inhalation anesthesia and were approved by the Sutter Institute for Medical Research IACUC. Animals were weighed on a weekly basis to ensure adequate weight gain. Animals were acclimated for a minimum of 4 days prior to the study procedure. No methods of restraint were used in this protocol. Sedation using a tiletamine/zolazepam combination, followed by mask induction with 100% oxygen and isoflurane, was used to facilitate intubation. Euthanasia techniques were based on the recommendations set forth in the *AVMA Panel on Euthanasia of Animals.* Urinalysis, hematology, and serum chemistry were done on day 0 and at termination. Animals were fasted prior to blood collections.

### Equipment

**FIG. 1.**
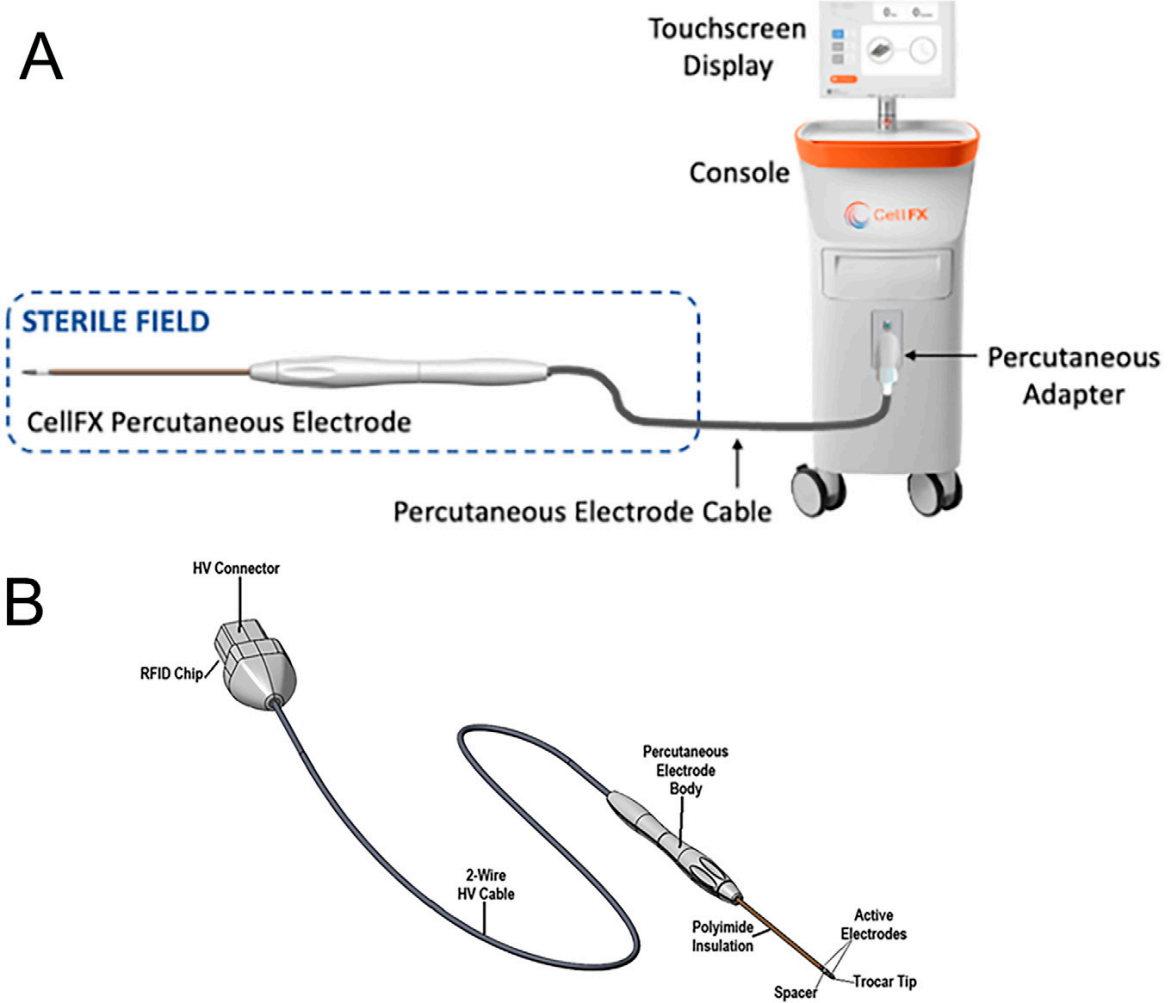
CellFX Percutaneous Electrode System.

**FIG. 2.**
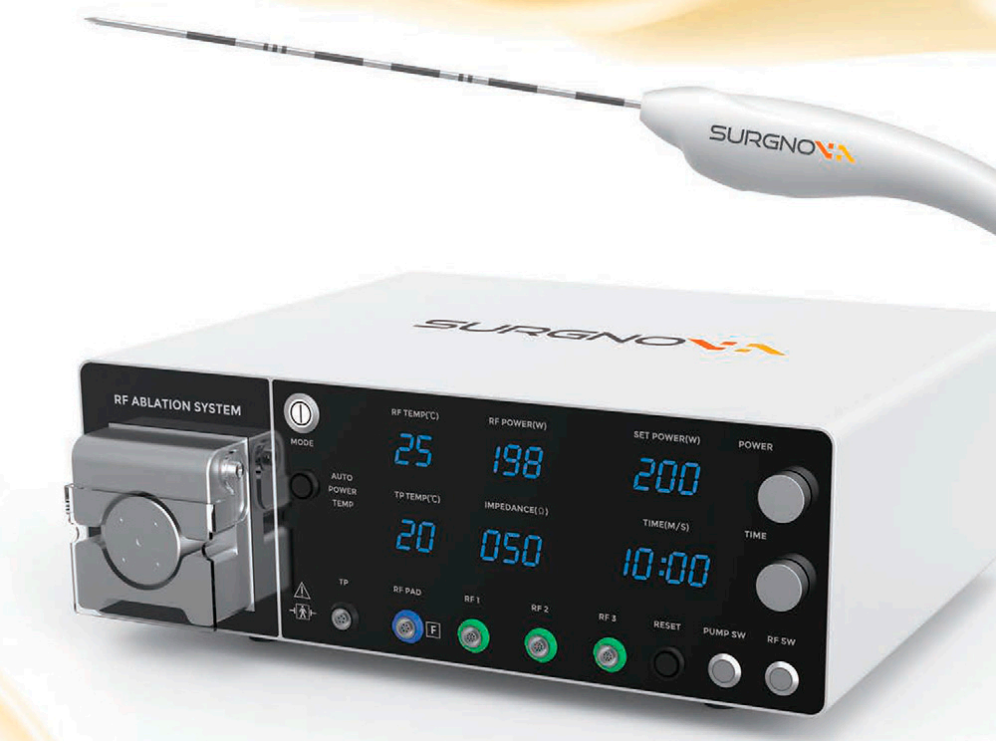
Dophi R150E Ablation System for generating RFA with R150 Electrode Kit, 19G. RFA, radiofrequency ablation.

The CellFX Percutaneous Electrode System was fabricated by Pulse Biosciences, Inc. and was composed of the CellFX console (pulse generator) and a percutaneous bipolar electrode (Fig. 1). The Dophi R150E was used with the Dophi R150 Electrode Kit, 19G, L 7 cm, exposure 15 mm, for the RF ablations (Fig. 2).

**FIG. 3.**
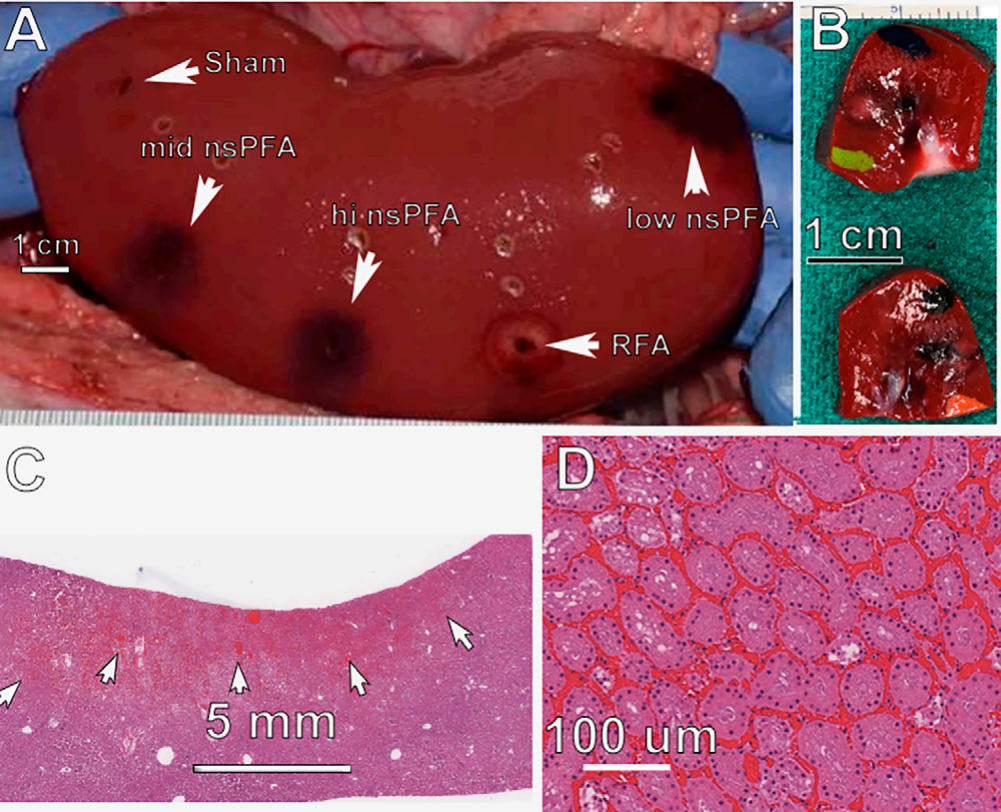
Day 0 Left porcine kidney **(A)** photographed posttreatment with indicated energies; **(B)** photo of fixed tissue near mid nsPFA treatment showing white TTC-stained ablation zone; **(C)** histological section from nsPFA ablation zone (between arrows and upper edge) stained with H&E; **(D)** higher magnification of ablation zone in “C.” H&E, hematoxylin and eosin; nsPFA, nanosecond pulsed field ablation; TTC, tetrazolium chloride.

### Randomization and control of bias

The distribution of the CellFX, sham CellFX, and untreated/naïve and blank sites was randomized for each animal. The veterinarian conducting gross necropsy, including treatment zone measurements and histopathological evaluations, was blinded regarding the treatment identity and energy setting.

### Study design

Animals were divided into two cohorts of three animals each for acute (day 0) and subacute (2 days). On day 0, randomized soft-tissue treatments were performed in the study animals using the CellFX and the Dophi RF. Three energy settings were used on the kidney, liver, and muscle. For exposure of the kidneys and liver for treatment, a laparotomy was performed. An initial midline incision was made along the cranial-caudal axis of sufficient length to allow access to both the liver and kidneys. Once the initial incision had been made, an electrocautery instrument was used to extend and deepen the incision through the skin, fat, muscle, and facia to expose the abdominal cavity. Retractors were used to further widen the laparotomy to allow access to the individual target organs. For the 2-day cohort, a sterile windowed drape was placed over the laparotomy to reduce the risk of accidental contamination of the aseptic field. For kidney treatments, the percutaneous electrode was inserted about 1.5 cm into the tissue; liver treatments inserted about 2.5 cm into the tissue. Once all the desired treatments had been completed in the liver and kidneys, all internal organs were returned to their original positions, and the laparotomy was closed using a combination of absorbable and nonabsorbable sutures, and in some cases, skin glue and/or staples were used, as per the facility’s standard operating procedure.

The animal was then moved into the ventral recumbent position, and the dorsum in the area to be treated was shaved and then cleaned using three alternating rounds of betadine and isopropanol. A sterile, windowed surgical drape was placed over the animal so that only the area undergoing treatment was exposed. A small incision was made in each treatment site with a scalpel, ensuring sufficient depth to reduce the pressure required to insert the electrodes through the dorsum and into the underlying musculature. The 1.4-cm-long active zone of the electrodes was inserted approximately 4 cm into the incision perpendicular to the surface of the skin. A piece of sterile tubing was placed over the shaft of the electrode to aid in consistent electrode insertion to this depth. Rocuronium was administered as necessary to reduce any muscle contractions. For sham treatments, the electrode was inserted and held in place for 125 s, corresponding to the longest treatment time tested in the protocol but no energy was delivered.

### Survival

Animals designated as part of the acute (day 0) cohort had blood and urine drawn immediately after completion of all muscle treatments, followed by IV tetrazolium chloride (TTC) injection. TTC is reduced by mitochondrial enzymes into a water-soluble compound in living cells that turns healthy tissue red. After TTC administration, the animal was euthanized. Animals designated as part of the 2-day cohort were allowed to recover from anesthesia. Two days after treatment, the animal was euthanized for necropsy and tissue harvest after blood and urine sample collection and TTC injection. TTC was administered intravenously at least 15 min prior to injection of the potassium chloride for euthanasia to allow for sufficient circulation time to ensure adequate staining.

### Necropsy

Photographs were taken of the closed incision site and all tissue treatment sites in situ and after harvesting. The left and right kidneys were excised whole from the abdominal cavity postmortem, photographed, and then grossed for lesion measurement and histological assessment. The liver was excised intact, photographed, and then grossed for lesion measurement and histological assessment. After the kidney and liver had been removed, the treated muscle sites were harvested along with the overlying skin in a single intact block on each side which contained all treatment sites for that side. The muscle tissue was separated into individual treatment blocks and then further grossed for lesion measurement and histological assessment. The excised blocks included at least 5 mm of nontreated tissue from the visible treatment in each direction to ensure the entirety of the treatment was contained in-block. Each block was bisected through the center of the treatment zone, and the dimensions of the lesion of each cut face were measured by the attending veterinarian using calipers. Photographs were taken capturing each measurement. After treatment zone measurement, each block was placed in a labeled cassette and submerged in a sample jar containing 10% neutral buffered formalin. Once fixed for at least 3 days, the formalin was poured out from the jar, the cassette was wrapped in gauze or paper towel soaked in formalin, and the tissue blocks were embedded. The resulting blocks were cut at 5 µm thickness, and the sections were stained by the hematoxylin and eosin (H&E) method.

## Results

### Ablation zone

After applying low, medium, and high energy densities (at least three times higher than clinical energy densities) for a duration of 25–125 s, the ablation zone in the three tissues was measured after TTC staining during gross necropsy (Table 1) and ranged from 20 to 57 mm in maximum depth (along electrode shaft). Width 1 (perpendicular to the shaft) and width 2 (also perpendicular to the shaft but with a 90° rotation) ranged from 11.1 to 27.5 mm. Each tissue block containing a lesion was first bisected vertically through the visible electrode insertion site, and each half was bisected again to form “quarters” and reveal the width of the ablation zone in both axes. Owing to this sectioning, the histological images of the ablation zone were formed by bringing together two half-zone images and that occasionally generated gaps at the center of the image. The ablation zone size differences between tissue types were probably because of the differences in tissue sensitivity to nsPFA. The maximum depth and width of the radiofrequency ablation (RFA) lesions are listed in Table 2 and are somewhat wider and less deep than nsPFA lesions despite the much larger energy density applied for RFA. Comparing the day 0 measurements with those of day 2, the ablation zone sizes were similar, but the contrast of the staining was greater on day 2 because of the slow time course of regulated cell death.

**Table 1.** nsPFA Ablation Zone Sizes in the Three Tissues as Grossly Measured with a Caliper

	Tissue	Energy density applied (J/mm^2^)	Max ablation depth (mm)	Max ablation width 1 (mm)	Max ablation width 2 (mm)
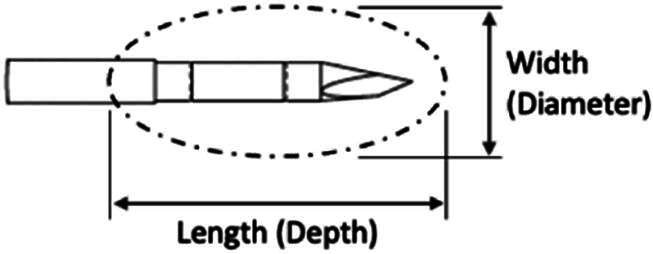	Kidney	1.1	20.9	11.1	11.1
	Liver	0.8	38.3	11.9	12.1
	Muscle	1.4	57.0	27.5	21.0

**Table 2.** RFA Ablation Zone Sizes in the Three Tissues

Tissue	Energy density (J/mm^2^)	Max ablation depth (mm)	Max ablation width 1 (mm)	Max ablation width 2 (mm)
Kidney	47	17.8	16.9	15.1
Liver	47	22.6	15.3	14.5
Muscle	47	29.2	24.2	21.4

### Kidney treatments

**FIG. 4.**
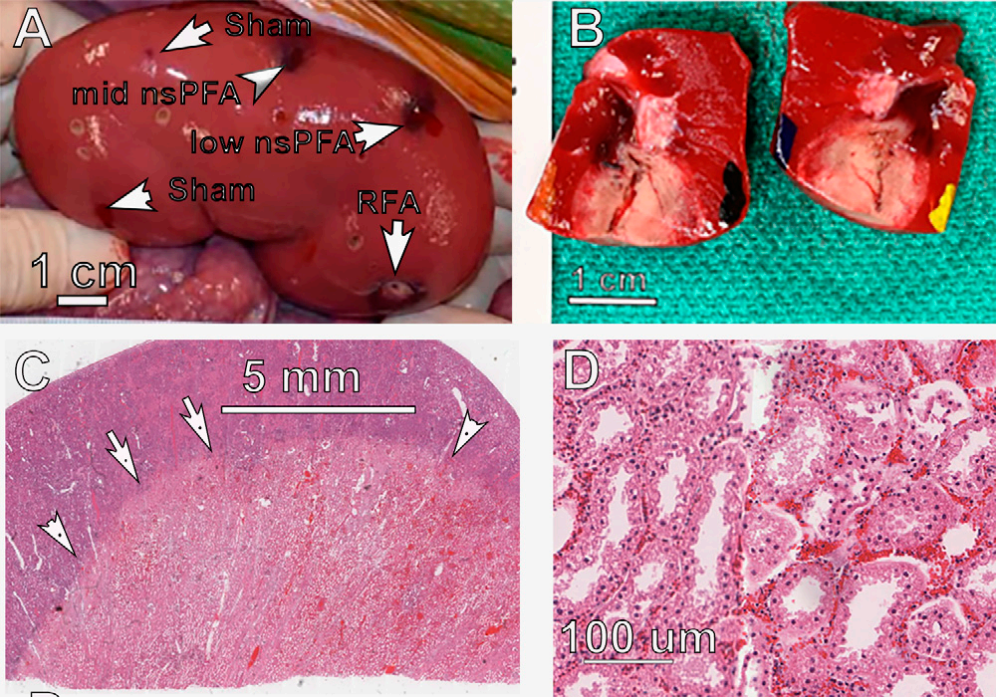
Day 2 posttreatment of porcine kidney. **(A)** Photo of kidney with sites of indicated applied energies; **(B)** fixed tissue around RFA treatment showing much larger treatment zone than observed with nsPFA and light discoloration of parenchyma with lack of TTC staining; **(C)** histological section from RFA ablation zone stained with H&E; **(D)** higher magnification of ablation zone in “C”.

The gross appearance of the nsPFA lesions was characterized by an easily discernible, generally well-demarcated area of dark/red/black and tan discoloration with areas of diminished TTC staining (Fig. 3A, B). The 2-day histology of the outer regions of the ablation zone exhibits tubular degeneration, and the deeper areas of the lesion exhibit hyperemia and hemorrhage with frank necrosis of tubules and glomeruli (Fig. 4C, D).

### Ablation zone histopathology

**FIG. 5.**
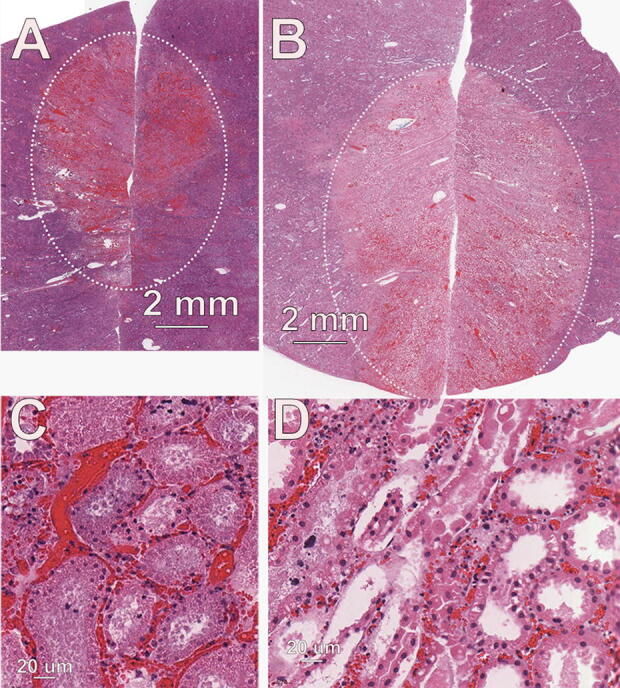
Ablation zone in porcine kidney. **(A)** nsPFA treatment of 0.9 J/mm^2^; **(B)** RFA treatment of 47 J/mm^2^; **(C)** higher magnification of ablation zone in “A”; **(D)** higher magnification of ablation zone in “B”. nsPFA, nanosecond pulsed field ablation; RFA, radiofrequency ablation.

Histological characteristics of the nsPFA treatments were similar among the three energy levels applied. There was a well-demarcated area of degeneration and necrosis of all cellular elements of the renal parenchyma, including tubular epithelial cells, all elements of glomeruli, and blood vessel cellular components (Fig. 3). The areas of frank necrosis were characterized by hyperacidophilia, cellular swelling, homogenization of cytoplasm, nuclear pyknosis, and often detachment of epithelial cells from their basement membranes. There were no observations of collagen degeneration. At the peripheral areas of the lesion, degenerate tubular epithelial cells exhibited cellular swelling, acidophilic cytoplasmic droplets, and cytoplasmic vacuolation. Hyperemia was a pronounced component of the treatment site lesions, and variable hemorrhage was observed at each site (Fig. 5).

Histological changes consistent with the RF-induced lesions were similar to those generated by nsPFA except for a somewhat smaller ablation zone, lower incidence of hemorrhage, a lower incidence of intravascular thrombus/fibrin, and the presence of subtle tinctorial changes suggestive of a thermally induced lesion. The RF ablation zone tended to be wider but not as deep as the nsPFA ablation zone.

### Liver treatments

The hepatic lesions were characterized by a well-demarcated dark/black discoloration region within the parenchyma with no contrast elucidated with the TTC. The dark appearance correlated to pronounced congestion/hyperemia. At 2 days after treatment, the lesions were very similar to those observed in the kidney. The RF lesions exhibited typical characteristics of a thermally induced lesion characterized by a well-demarcated region of discoloration consisting of a larger central area of tan discoloration because of thermal denaturation and a total lack of staining with TTC.

### Ablation zone histopathology

**FIG. 6.**
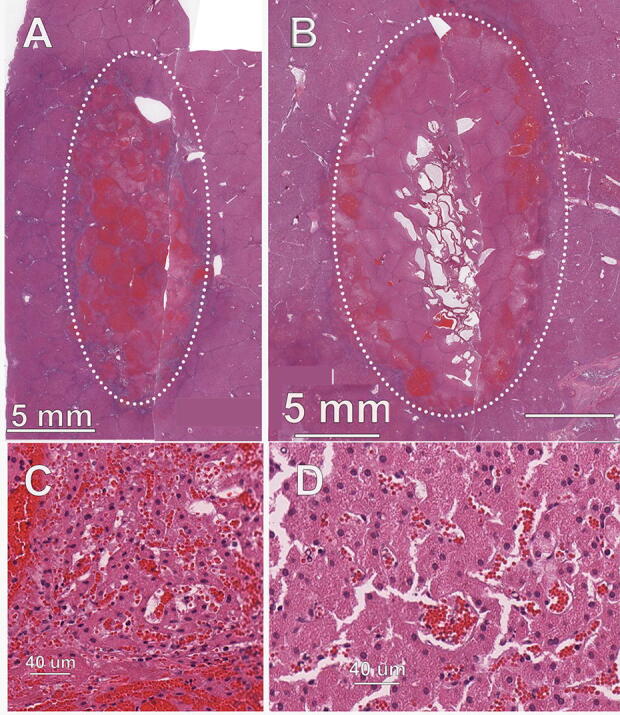
Ablation zone in porcine liver. **(A)** nsPFA treatment of 0.7J/mm^2^; **(B)** RFA treatment using 47 J/mm^2^; **(C)** higher magnification of ablation zone in “A”; **(D)** higher magnification of ablation zone in “B”.

NsPFA treatment sites exhibited hyperemia with hemorrhage and degeneration and necrosis of all cellular elements of the hepatic parenchyma, including hepatocytes, ductular epithelial cells, and blood vessel cellular components (Fig. 6). The necrotic cells were characterized by hyperacidophilia, cellular swelling, homogenization of cytoplasm, and nuclear pyknosis. RF lesions were very similar to the nsPFA lesions, with the exception of tinctorial changes consistent with a thermal lesion, including occasional areas of degeneration of collagen.

### Muscle treatments

**FIG. 7.**
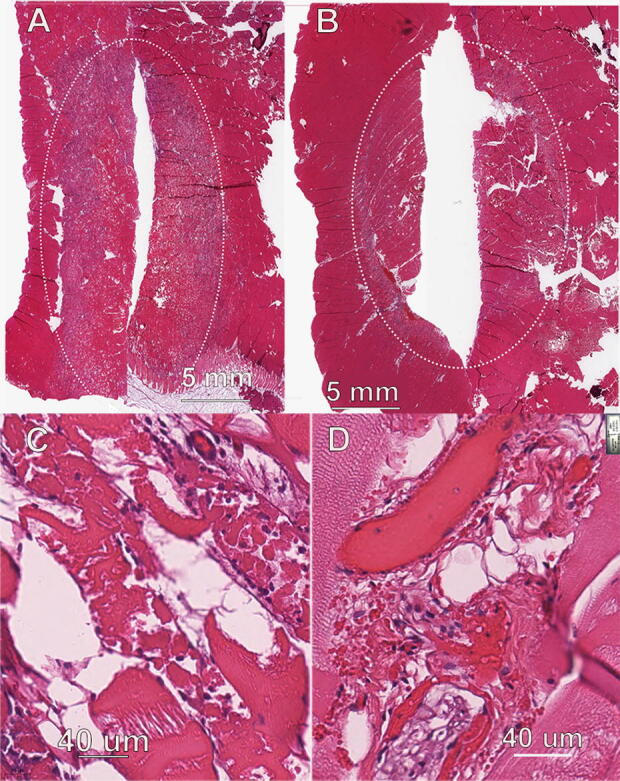
Ablation zone of porcine muscle. **(A)** nsPFA treatment of 1.1 J/mm^2^; **(B)** RF treatment of porcine muscle using 47 J/mm^2^. The discontinuity where the two thin sections meet is because of a sectioning artifact; **(C)** higher magnification of ablation zone in “A”; **(D)** higher magnification of ablation zone in “B”.

Day 0: easily discernible, well-demarcated regions of light discoloration because of decreased TTC staining. Day 2: treatment site lesions were easily discernible and well demarcated, consisting of a larger central area of dark red discoloration and a narrow peripheral ring of light tan/pink discoloration because of diminished TTC staining (Fig. 7).

The nsPFA treatment site lesions consisted of a generally well-demarcated area of degeneration and necrosis of myocytes and blood vessels. The necrotic myocytes exhibited cellular swelling, hyperacidophilia, and homogenization of the sarcoplasm. Edema was characterized as an increase in clear space between individual myocytes and secondary bundles. There were no observations of collagen degeneration. Low numbers of neutrophils were observed within the interstitial areas immediately adjacent to necrotic myocytes and a low number of macrophages were also a component of the inflammatory infiltrate. Unlike the other two tissues, the width of the ablation zones of the nsPFA and RF treatments did not differ significantly in size. RF-induced lesions appeared very similar to the nsPFA lesions, with the exception of tinctorial changes of striated myocytes consistent with thermal effects.

## Discussion

The objective of this study was to evaluate the performance of the CellFX Percutaneous Electrode for use with nsPFA energy delivered to kidney, liver, and skeletal muscle at day 0 and 2 days after treatment in comparison with RFA. The CellFX system successfully achieved degeneration and necrosis of all treatment sites in all target tissues. No evidence of thermal effects or collagen degeneration was found at any of the nsPFA treatment sites. Overall systemic tolerability was evidenced by the absence of changes in urinalysis and serum chemistry before and after treatments.

The sizes of the ablation zones in each tissue vary considerably, with muscle exhibiting the largest ablation zone both in width and depth. Muscle cells are very sensitive to nsPFA and are ablated at lower field strengths than the other tissues. This is probably because of their sensitivity to the increase in intracellular Ca^2+^, which is normally used to control contractions. The large Ca^2+^ increase resulting from nsPFA overwhelms their normal capacity to pump Ca^2+^ out, and the permeabilization of their mitochondria reduces ATP production needed to power the pumps.

### Advantages of nsPFA

NsPFA has many advantages over RF ablation. RF ablation heats the tissue to denature proteins and acellular structures, and the spread of heat in tissue risks damaging nerves and other sensitive elements. NsPFA is nonthermal and does not impact acellular tissue, such as collagen or cartilage, so it offers significant safety advantages over thermal modalities by allowing surgeons to ablate near and into vessels and acellular structures without concern of permanent damage. NsPFA ablates much faster than thermal modalities and has been shown to spare nerves from any permanent damage, even when treated directly, unlike thermal modalities.

### Limitations of this study

A single treatment for each energy setting limits the measure of reproducibility. The size of the ablation zone is limited by the field strength required to ablate tissue with nsPFA. The maximum CellFX pulse amplitude is 15 kV, and the field strength required to ablate tissues is in the range of 20–30 kV/cm. Therefore, multiple applications of the electrode moved to different positions will be needed to ablate tissue regions larger than the ablation zones reported here.
